# Xenogeneic Graft-Versus-Host Disease in Humanized NSG and NSG-HLA-A2/HHD Mice

**DOI:** 10.3389/fimmu.2018.01943

**Published:** 2018-08-30

**Authors:** Grégory Ehx, Joan Somja, Hans-Jörg Warnatz, Caroline Ritacco, Muriel Hannon, Loïc Delens, Gilles Fransolet, Philippe Delvenne, Joséphine Muller, Yves Beguin, Hans Lehrach, Ludovic Belle, Stéphanie Humblet-Baron, Frédéric Baron

**Affiliations:** ^1^Hematology Research Unit, Groupe Interdisciplinaire de Génoprotéomique Appliquée (GIGA)-I^*3*^, University of Liège, Liège, Belgium; ^2^Department of Pathology, CHU of Liège, Liège, Belgium; ^3^Otto Warburg Laboratory Gene Regulation and Systems Biology of Cancer, Max Planck Institute for Molecular Genetics, Berlin, Germany; ^4^Department of Medicine, Division of Hematology, CHU of Liège, Liège, Belgium; ^5^Alacris Theranostics GmbH, Berlin, Germany; ^6^Translational Immunology Laboratory, VIB, Leuven, Belgium; ^7^Department of Microbiology and Immunology, KU Leuven, Leuven, Belgium

**Keywords:** GVHD, xenogeneic, NSG, NSG-HLA-A2/HHD, TCR receptor, TCRβ repertoire, RNA sequencing, graft-versus-leukemia

## Abstract

Despite the increasing use of humanized mouse models to study new approaches of graft-versus-host disease (GVHD) prevention, the pathogenesis of xenogeneic GVHD (xGVHD) in these models remains misunderstood. The aim of this study is to describe this pathogenesis in NOD/LtSz-Prkdc^scid^IL2rγ^tm1Wjl^ (NSG) mice infused with human PBMCs and to assess the impact of the expression of HLA-A0201 by NSG mice cells (NSG-HLA-A2/HHD mice) on xGVHD and graft-versus-leukemia (GvL) effects, by taking advantage of next-generation technologies. We found that T cells recovered from NSG mice after transplantation had upregulated expression of genes involved in cell proliferation, as well as in TCR, co-stimulatory, IL-2/STAT5, mTOR and Aurora kinase A pathways. T cells had mainly an effector memory or an effector phenotype and exhibited a Th1/Tc1-skewed differentiation. TCRβ repertoire diversity was markedly lower both in the spleen and lungs (a xGVHD target organ) than at infusion. There was no correlation between the frequencies of specific clonotypes at baseline and in transplanted mice. Finally, expression of HLA-A0201 by NSG mice led to more severe xGVHD and enhanced GvL effects toward HLA-A2^+^ leukemic cells. Altogether our data demonstrate that the pathogenesis of xGVHD shares important features with human GVHD and that NSG-HLA-A2/HHD mice could serve as better model to study GVHD and GvL effects.

## Introduction

Allogeneic hematopoietic stem cell transplantation (allo-HCT) offers potential curative treatment for a wide range of hematological disorders (1). In patients with hematological malignancies, tumor eradication depends both on the conditioning regimen given before allo-HCT, and on graft-versus-leukemia (GvL) effects mediated by donor immune cells (mainly T cells) contained in the graft (2–4). Unfortunately, donor T cells can also attack healthy tissues of the recipient, causing graft-versus-host disease (GVHD), a life-threatening complication of allo-HCT (5–7).

GVHD pathogenesis has been mostly studied in mouse-to-mouse models of transplantation (8–12). However, these models of GVHD have some caveats such as: (1) fixed genetic and immunologic disparities between donor and recipient within a determined murine GVHD model; (2) the uniform use of young adult mice (in contrast to the increasing use of allo-HCT in older patients/donors); (3) the fact that mice are housed from birth to death under pathogen-free conditions while donors and patients are exposed before and after transplantation to pathogens than can affect their immune responses (13, 14); and (4) important divergences between murine and human immunity (15–17).

In the last decades, humanized murine models of xenogeneic GVHD (xGVHD) have been developed (18). Among these, the “NSG xGVHD murine model” consisting of injection of human peripheral blood mononuclear cells (hPBMC) into NOD/LtSz-Prkdc^scid^IL2rγ^tm1Wjl^ (NSG) mice is increasingly used (19–30). NSG mice combine the lack of T-cell and B-cell activity of the SCID mice and the reduced macrophage activity of the NOD mice with the absence of NK activity due to the absence of the expression of the IL2rγ. Consequently, low doses of hPBMC consistently engraft in these mice even without prior irradiation, although irradiation significantly increases xGVHD severity (19–22).

In comparison to mouse-to-mouse models of GVHD, the NSG “humanized” model has some advantages such as the use of human cells to induce and control xGVHD, the possibility to use donors with a high genetic diversity, and the possibility to use older donors previously exposed to various pathogens. However, the pathogenesis of xGVHD induced by injection of hPBMCs in NSG mice has been only partly elucidated. It has been established that human T cells are able to recognize murine xeno-antigens presented by murine major histocompatibility complex (MHC) (31). Further, human CD28 receptor is able to interact with murine B7.2 molecules thus providing a second signal for human T cells (32). In accordance with these prior observations, it has been elegantly demonstrated that recognition of murine MHC by human T cells plays an important role in the pathogenesis of xGVHD in NSG mice. Specifically, xGVHD following injection of hPBMC was delayed in NSG mice lacking expression of MHC class II expression (NSG-Ab° mice) (20), while NSG mice lacking MHC class I expression were resistant to xGVHD induced by injection of hPMBC (20). These observations suggest that recognition of MHC class I murine molecules by human CD8^+^ T cells is preponderant in xGVHD. In accordance with these observations, Søndergaard et al. nicely demonstrated that human CD8^+^ T cells were sufficient and required for xGVHD induction, while injection of purified CD4^+^ T-cells failed to induce xGVHD in NSG mice (22). Further, these authors demonstrated that the CD28-B7.2 second signal was required for human T-cell proliferation and xGVHD induction in NSG mice infused with hPBMC (22). Interestingly, analyses by classical spectratyping showed that TCRβ repertoire was diverse after hPBMC transplantation in NSG mice (21, 22), suggesting that there was a high proportion of non-TCR-specific driven T-cell proliferation in the pathogenesis of xGVHD.

Here, we took advantage of recent techniques (such as RNA sequencing (RNAseq), multicolor flow cytometry and deep TCRβ sequencing) to further describe the pathogenesis of xGVHD induced by injection of hPBMCs in NSG mice. In addition, we compared xGVHD pathogenesis in classical NSG mice and in NOD-scid IL-2Rγ^null^, HLA-A2/HHD (NSG-HLA-A2/HHD) mice (expressing HLA-A0201 molecules and the human β2 microglobuline in addition to mouse MHC class 1 and class 2 molecules) (33, 34) in which xGVHD could better mimic the clinical setting of allo-HCT in which human T cells are activated through interaction with human, and not mouse, MHC molecules.

## Material and methods

### PBMCs isolation and HLA-A typing

Peripheral blood was obtained from healthy volunteers (aged 23–35 years old) after they signed a written informed consent approved by our Ethical Committee. PBMCs were harvested from peripheral blood by Ficoll-Paque (GE Healthcare, Freiburg, Germany) gradient centrifugation. HLA-A typing was performed by flow cytometry using an anti-HLA-A2 antibody (see next sections for flow cytometry protocols) while the presence of HLA-A0201 was confirmed by PCR.

### Induction and assessment of xGVHD in NSG mice

NSG or NSG-HLA-A2/HHD mice (The Jackson laboratory, Bar Harbor, ME), aged from 8 to 10 weeks, were irradiated with 2.5 Gy and given 24 h later an i.v. injection of 1 × 10^6^ (in some experiments) or 2 × 10^6^ (in most experiments) hPBMCs to induce xGVHD. GVHD severity was assessed by a scoring system that incorporates four clinical parameters: weight loss, posture (hunching), mobility and anemia as previously reported (24). Each parameter received a score of 0 (minimum) to 2 (maximum). Mice were assessed for GVHD score thrice weekly and monitored daily during the experiments. Mice reaching a GVHD score of 6/8 were sacrificed in agreement with the recommendation of our ethical committee. Final scores for dead animals reaching the ethical limit score were kept in the data set for the remaining time points (last value carried forward). All experimental procedures and protocols used in this investigation were reviewed and approved by the Institutional Animal Care and Use Ethics Committee of the University of Liège, Belgium. The “Guide for the Care and Use of Laboratory Animals,” prepared by the Institute of Laboratory Animal Resources, National Research Council, and published by the National Academy Press, was followed carefully.

### RNA sequencing and bioinformatics analysis

Five NSG and five NSG-HLA-A2/HHD mice were transplanted with PBMCs from four non-HLA-A2 different donors (2 × 10^6^ PBMCs iv, 24 h after 2.5 Gy TBI), 3 out of 5 mice receiving the PBMCs from a single donor for each mouse while the two remaining animals received the PBMCs from the same donor. One million of CD3^+^ cells were sorted from PBMCs of each donor by flow cytometry and preserved at −80°C in Tripure (Roche) while another million of sorted T cells was stimulated *in vitro* with CD3/CD28 dynabeads (bead:cell ratio 1:1, Invitrogen, Waltham, MA) in X-VIVO 15 (Lonza, Verviers, Belgium). After 4 days of culture, 1 million of T cells was collected from the stimulated cells and cryopreserved in Tripure. At day 7 post-transplantation, mice were sacrificed and 1 million of human CD3^+^ T cells were sorted from their spleen (in all but one NSG-HLA-A2/HHD mice which died due to irradiation). All sorted T cells were preserved at −80°C in Tripure until the day of RNA extraction. Total RNA was isolated (RNeasy Mini Kit, Qiagen, Venlo, The Netherlands) and used for poly(A) selection and Illumina Truseq stranded library preparation following manufacturer's instructions. Samples were sequenced on the Illumina NextSeq500 to an average depth of 23.8 × 10^6^ 76-bp reads per sample. Reads alignment, processing and counting were performed with the “RNA-seq Alignment 1.1.0” app from Illumina BaseSpace, reads were aligned to the human genome (RefSeq UCSChg19) using STAR 2.5.0b, novel transcript assembly and default settings. The average read alignment rate was 99.2%. Differential expression, principal component analyzes and hierarchical clustering heatmaps were computed using DESeq2 1.16.1 in R (version 3.4.1) (35) on genes having at least one read in at least one sample (19,846 out of 26,364 genes). Genes significantly up- or down-regulated were defined as genes with a change in expression of at least 2-fold or 0.5-fold, respectively, and a FDR-adjusted *p*-value of ≤ 0.05. Gene set expression analyzes were performed in GSEA software v3.0 (36) using the FPKM data, generated by the RNA-seq Alignment app, as input (including all genes having at least one read in at least one sample). The HALLMARK gene sets (accessed on 04 September 2017) were used to analyze the pathways differentially expressed between the four different conditions. The costimulation by CD28 pathway was analyzed using the gene set of the same name from REACTOME (M17386, accessed on 18 September 2017), the TCR signaling was analyzed using the signature of 165 genes described by Datlinger et al. (37) (Supplemental Table 5), the Th1/Th2 pathway by the BIOCARTA_TH1TH2_Pathway (M6705, accessed on 15 January 2018), the Th17 differentiation by the RORγt and RORα signature genes set (38) (Supplemental Table 7) and the Aurora kinase A by the PID_AURORA_A_PATHWAY (M242, accessed on 30 January 2018). Analyses were performed with 1,000 permutations (phenotype type) for *p*-value calculation. Default parameters were used. Gene sets with FDR (*q*-value) ≤ 0.25 were considered statistically significantly differentially expressed.

### Flow cytometry

At the time of necropsy, peripheral blood (PB), spleen, bone marrow (BM), liver and lungs were harvested and analyzed by flow cytometry. PB was depleted of erythrocytes using RBC lysis buffer (eBioscience, San-Diego, CA) according to manufacturer's instructions. Splenocytes were obtained by crushing the spleen, and BM cells by flushing femurs and tibiae. Lungs and liver infiltrating cells where obtained by mincing and incubating the organs for 1 h in HBSS + 50 μg/ml DNase 1 (Roche, Basel, Switzerland) + 1 mg/ml collagenase A (Roche). Digestions were stopped by washing twice with PBS+EDTA 10 mM, pH = 7.3, and mononuclear white blood cells where collected by ficoll gradient centrifugation. Cells from all organs were washed twice with PBS+3% FBS and were counted with a Sysmex XS-800i^®^ before processing with antibodies staining. The following antibodies specific for human antigens were used: anti-CD45-APC (HI30); anti-CD45-PECy7 (HI30); anti-CD45-PerCP (2D1, BD); anti-CD4-eFluor450 (RPA-T4); anti-CD4-PerCP (SK3, BD); anti-CD8-PECy7 (SK1, SONY); anti-CD8-BV510 (RPA-T8, Biolegend, San-Diego, CA); anti-CD45RA-BV510 (HI100, BD); anti-CD27-PE (M-T271, BD); anti-CD62L-APC-eFluor780 (DREG56), anti-CD25-BV421 (M-A251, BD); anti-HLA-DR-APC-eFluor780 (LN3); anti-GRANZYME B-PE (GB11, BD); anti-FOXP3-AlexaFluor488 (206D, Biolegend); anti-KI67-AlexaFluor647 (B56, BD); anti-IL-17-APC (eBio64DEC17); anti-IFNg-PECy7 (4S.B3); anti-TNF-α-APCCy7 (Mab11, SONY); anti-IL-4-PE (MP4-25D2, SONY); anti-PD-1-APC-eFluor780 (eBioJ105); anti-phosphoSTAT5-AlexaFluor647 (pY694, BD); and the following antibody specific for mouse antigens was used: anti-CD45-FITC (30-F11, BD) (all from eBioscience unless indicated otherwise). Cells (1.5–2 × 10^6^ cells/sample), resuspended in 50 μl of PBS+3%FBS, were incubated with surface antibodies for 20 min at 4°C in the dark and washed twice with PBS+3% FBS. Intracellular staining for FOXP3, KI67, GZMB and cytokines was performed by using the FoxP3 Staining Buffer Set (eBioscience). For intracellular cytokine staining, spleen homogenates were stimulated for 4 h in RPMI supplemented with 10% FBS, penicillin (100 U/mL), streptomycin (10 mg/mL) and in presence of PMA/ionomycin, brefeldin A and monensin (Cell Stimulation Cocktail + Protein Transport Inhibitors, eBioscience). For pSTAT5 staining, the PerFix EXPOSE reagent kit (Beckman Coulter, Fullerton, CA) was used as previously reported (39). Total cell count of human CD45^+^ cells in blood were calculated based on the absolute number of white blood cells (counted by using a Sysmex XS-800i cell counter) and on the human cell chimerism [frequency of human CD45^+^ cells among the total white blood cell population (%_human_CD45^+^+%_mouse_CD45^+^)]. Data were acquired on a FACS Canto II flow cytometer (Becton Dickinson) and analyzed with the Flowjo software 7.0 (Tree Star Inc., Ashland, OR). In gating strategies, regulatory T cells (Treg) were defined as CD4^+^CD25^+^FOXP3^+^ while remaining CD4^+^ T cells were termed conventional T cells (Tconv). Naïve T cells were defined as CD45RA^+^CD27^+^, effector T cells (TE) as CD45RA^−^CD27^−^, effector memory T cells (TEM) as CD45RA^−^CD27^+^CD62L^−^, and central memory T cells (TCM) as CD45RA^−^CD27^+^CD62L^+^.

### TCR repertoire diversity

Spleens or lungs from 10 NSG or 10 NSG-HLA-A2/HHD mice were pooled into a NSG and a NSG-HLA-A2/HHD pool and were processed to stain human CD45, CD3, CD4 and CD8 antigens for flow cytometry sorting. A total of one million of CD45^+^CD3^+^CD4^+^ and of CD45^+^CD3^+^CD8^+^ T cells were sorted from each organ and each mouse strains, and from the hPBMC donor (collected the day of transplantation). DNA was extracted immediately from these cells by using the PureLink Genomic DNA Mini Kit (Invitrogen); 125 μg from each samples were used for T-cell receptor β chain (TCRβ) deep sequencing performed by Adaptive Biotechnologies (Seattle, WA, USA).

### Histology and immunochemistry

Lungs and liver from mice sacrificed at day 14 after transplantation were harvested, washed with PBS, fixed in 10% formalin (Sigma Aldrich) and routinely processed for paraffin embedding. Five μm sections were stained with hematoxylin-eosin (H&E) for histological examination. Immunostaining was performed on 5-μm sections using a BenchMark XT autostainer (Ventana Medical Systems, Tucson, AZ, USA) for human CD3 (clone PS1; Novocastra/Leica, Newcastle Upon Tyne, UK). Slides were assessed by using a semiquantitative scoring system for abnormalities known to be associated with GVHD, adapted from Blazar et al. (40). Fourteen parameters were scored for lungs (perivascular inflammation, peribronchiolar inflammation, interstitial infiltrate endothelitiis, airway epithelial apoptosis, airway epithelial detachment, alveolar edema, alveolar debris, alveolar damage, fibrosis, hemorrhage eosinophilic crystals, bronchiolar epithelial hyperplasia, and bronchiolar epithelial mucous production) and 16 for the liver (portal tract expansion by inflammatory cell infiltrate, lymphocytic infiltrate of bile ducts, bile periductal inflammatory infiltrate, bile duct hyperplasia, ductopenia, bile duct epithelial cell apoptosis, bile duct epithelial cell sloughing, bile duct epithelial hyalinosis, vascular endotheliitis, parenchymal apoptosis, hepatocyte degeneration/ballooning, parenchymal microabscessses, parenchymal mitotic figures, hepatocellular cholestasis hepatocellular steatosis, and fibrosis). Each parameter was scored as follows: 0 indicates normal; 0.5, focal and rare; 1, focal and mild; 2, diffuse and mild; 3, diffuse and moderate; and 4, severe.

### Serum cytokine levels

The concentration of human cytokines was determined in mice serum, after 2-fold dilution, using a custom Magnetic Luminex Performance Assay (R&D Systems, Minneapolis, MN, USA). The experiments were performed according to the manufacturer's recommendation, and results were acquired on Bio-Plex System and analyzed with Bio-Plex Manager Software 4.0 (Biorad Laboratories) as previously reported (41).

### GvL effects and bioluminescence imaging

For GvL effect assessment, mice were co-injected with 1 million luciferase-expressing THP-1 cells and 1 million hPBMC from non-HLA-A0201 donor after 2.5 Gy TBI. Twenty-one days later, three non-transplanted NSG mice and all transplanted NSG and NSG-HLA-A2/HHD mice were further injected subcutaneously in the left flank with 1 million of THP-1 cells in matrigel. Bioluminescence analyses were made as previously reported (42). In brief, mice were injected i.p. with 3 mg of D-luciferin (Promega, Madison, WI, USA) in PBS and imaged 12 min later. Tumor growth was evaluated by measuring bioluminescence of THP-1 cell line transfected to express the luciferase reporter gene by using the bioluminescent IVIS imaging system (Xenogen-Caliper, Hopkinton, MA). Mice were anesthetized using isoflurane (2.5% vaporized in O2). For analysis, total photon flux (photons per second) was measured from a fixed region of interest over the entire abdomen using Living Image 2.60.1 and IgorPro software (Wavemetrics).

### Statistical analyses

The Mann-Whitney test was used to compare flow-cytometry data between two different groups. Comparisons between GVHD score curves were made using the 2-way ANOVA test. Survival curves were modeled using the Kaplan-Meier methods. Comparisons between groups were made with the log-rank test. Multivariate Cox models adjusted for weight, female PBMC donor to male mouse, and age of mice at transplantation were also performed. In order to determine whether the survival differences between NSG and NSG-HLA-A2/HHD mice injected with human PBMC were consistent with different donors, meta-analyses of Hazard ratio obtained with each different donor were performed using fixed effects models. *P*-values < 0.05 were considered as statistically significant and all *P*-values were 2-sided. Statistical analyses were carried out with Graphpad Prism 5.0 (Graphpad Software, San Diego, CA, USA) or with NCSS 11 (NCSS Statistical Software, LLC, Kaysville, UT, USA).

### Data availability

RNA-Seq data have been deposited in the ArrayExpress database at EMBL-EBI (www.ebi.ac.uk/arrayexpress) under accession number E-MTAB-6865. The datasets supporting the conclusions of this article are included within this article and its additional files.

## Results

### Comparison of the transcriptomes of T cells at baseline, after *in vitro* activation and after transplantation in NSG mice

To gain insight into the mechanisms of T-cell expansion in NSG and NSG-HLA-A2/HHD mice, we performed a RNA-sequencing of T cells sorted from mouse spleens 7 days post-transplantation. As controls, we analyzed the transcriptome of T cells sorted from the same PBMCs used to transplant the animals, which were either not stimulated (resting condition) or *in vitro*-stimulated with CD3/CD28 beads (CD3CD28 condition).

**Figure 1:**
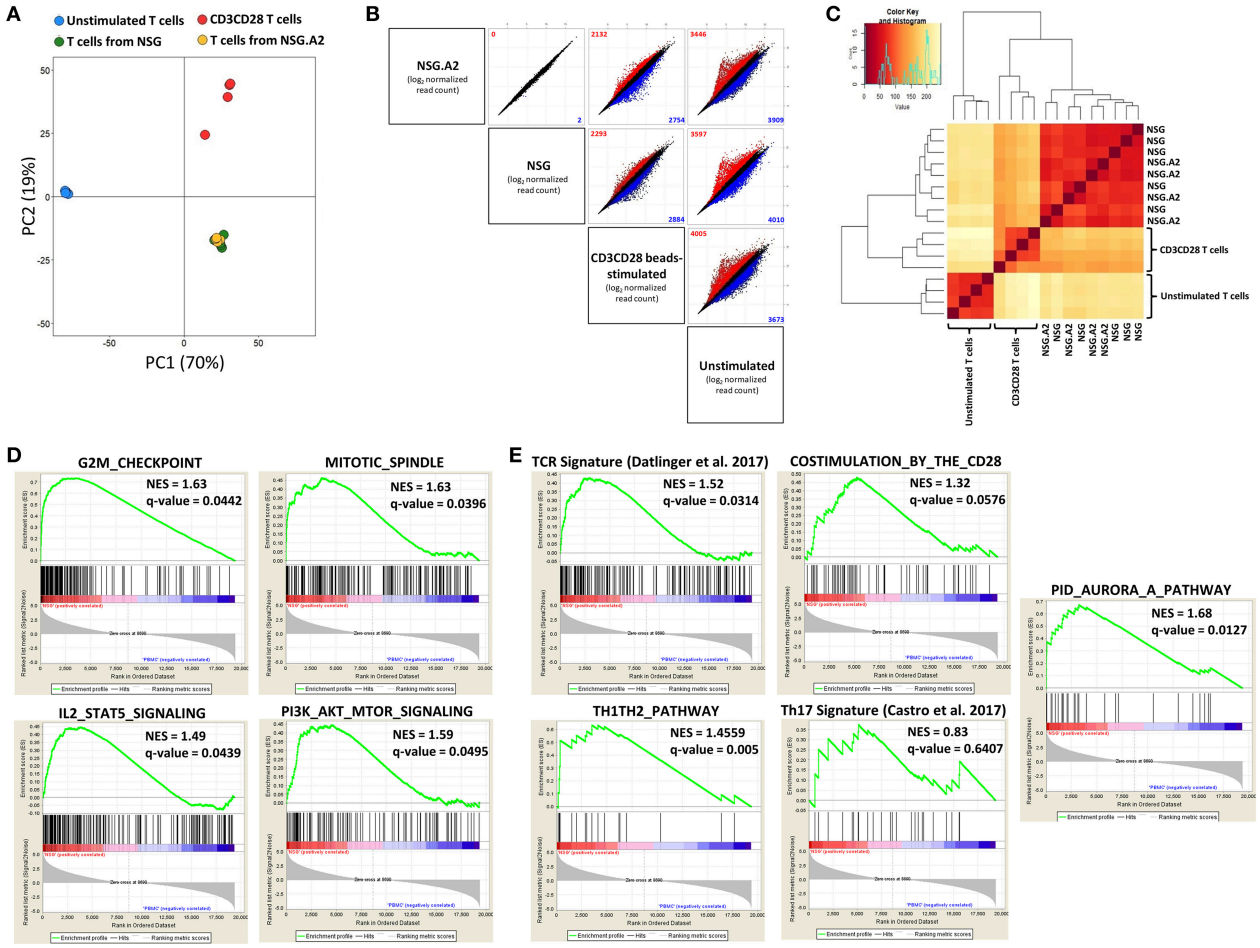
RNA sequencing of T cells isolated from spleen of NSG and NSG-HLA-A2/HHD mice. RNA-sequencing was performed on T cells sorted from the spleen of NSG and NSG-HLA-A2/HHD (NSG.A2 in figure) mice at day 7 post-transplantation, as well as on unstimulated T cells and CD3/CD28-stimulated T cells (*n* = 4–5 in each condition). **(A)** Principal-component analysis performed using all genes having at least one read in at least one sample. **(B)** Gene expression (average of log_2_ normalized read counts) in every pairwise comparison between the different conditions. Colors indicate significant (FDR ≤ 0.05) upregulation (of at least 2-fold; red) or downregulation (of at least 0.5-fold; blue) of expression in the comparison of the condition represented on the left vs. the condition represented on the bottom of the panel. Numbers in plots indicate total genes upregulated (red) or downregulated (blue). **(C)** Heatmap of hierarchical clustering of the different samples. **(D)** Gene set enrichment analyzes (GSEA) of the NSG vs. unstimulated T cells comparison. **(E)** GSEA of the NSG vs. unstimulated T cells comparison for TCR signaling, costimulation, Th1Th2 differentiation, Th17 differentiation by the RORγt and RORα signature genes set and Aurora kinase A pathway comparisons.

While the gene expression profile of T cells from NSG and NSG-HLA-A2/HHD mice were virtually identical, the greatest changes of expression were observed between resting T cells and T cells from all other conditions (Figures 1A,B). These differences were confirmed by a hierarchical heatmap showing that all mice samples clustered together and were more closely related to CD3/CD28 than to unstimulated samples (Figure 1C). These observations suggest that a main source of variation of T cells after injection in mice is their activation through TCR and CD28 pathways.

In order to confirm this hypothesis, we analyzed the variation of specific pathways by gene set enrichment analyzes (GSEA) in the NSG vs. unstimulated comparison. The HALLMARK matrix of gene sets highlighted that 30 out of 50 gene sets were significantly enriched in T cells from NSG mice (Supplemental Table 1). Among these pathways, five were linked to proliferation regulation (Figure 1D), six were associated with metabolism, five with immune responses and four with signaling pathways. Interestingly, the PI3K/AKT/mTOR (detailed in Supplemental Table 2) and IL-2/STAT5 (detailed in Supplemental Table 3) pathways were both upregulated, supporting the hypothesis that T cell proliferation in NSG mice is driven by IL-2 in addition to TCR stimulation and co-stimulation signals. Comparable results were obtained in the NSG-HLA-A2/HHD vs. unstimulated T cells comparison (Supplemental Table 4).

As the HALLMARK matrix does not include gene sets for TCR and costimulation pathways, we used a TCR signaling signature recently described (37) (Supplemental Table 5), and the co-stimulation by CD28 family gene set from REACTOME. As hypothesized, we observed significant enrichment of genes involved in each TCR and co-stimulation signaling after injection in mice (Figure 1E). We next assessed whether T-cell transplantation in NSG mice induced a Th polarization by comparing the profile of genes expressed in NSG vs. unstimulated T cells. We observed an upregulated expression of Th1 genes (IL2RA, IL12RB2, INFG, IL2, IL12RB1, and IL18) as well as of the Th2 gene IL4 in T cells recovered from the spleens while there was no upregulation of Th17 genes (Figure 1E and Supplemental Tables 6,7). Recently, Aurora Kinase A (AURKA) has been identified as promising target in the prevention of GVHD (43). Therefore, we assessed whether this pathway was altered in T cells expanding in NSG mice. As showed in Figure 1E, the AURKA pathway was highly upregulated in NSG mice, further supporting the physiological relevance of the NSG model for the study of GVHD.

GSEA analysis of the NSG vs. CD3CD28 samples showed that only the cholesterol homeostasis gene set of the HALLMARK matrix was significantly differentially expressed (upregulated in CD3CD28 samples). Regarding the other assessed gene sets, we found no significant difference of expression between both conditions for AURKA pathway, Th17 differentiation and co-stimulation signaling while TCR signaling and TH1TH2 differentiation were found to be significantly upregulated (FDR = 0.168 and 0.138, respectively) in NSG mice (data not shown).

In summary, these data show that, in comparison to baseline, T cells recovered from the spleen of mice 7 days after transplantation had upregulated expression of genes involved in cell proliferation, as well as in TCR, co-stimulatory, IL-2/STAT5, mTOR, AURKA, and Th1 signaling.

### Characterization of T cells recovered from mice 14 days after hPBMC infusion

Median proportion of T cells among human CD45 cells in the spleen on day 14 after transplantation were 43% (34–45%) in NSG and 47% (33–59%) in NSG-HLA-A2/HHD mice in case of non-HLA-A2 donor (*P* = 0.35) and 54% (43–58%) in NSG and 64% (53–70%) in NSG-HLA-A2/HHD mice in case of HLA-A2 donor (*P* = 0.051).

#### NSG and NSG-HLA-A2/HHD mice infused with NON-HLA-A2 hPBMC

**Figure 2:**
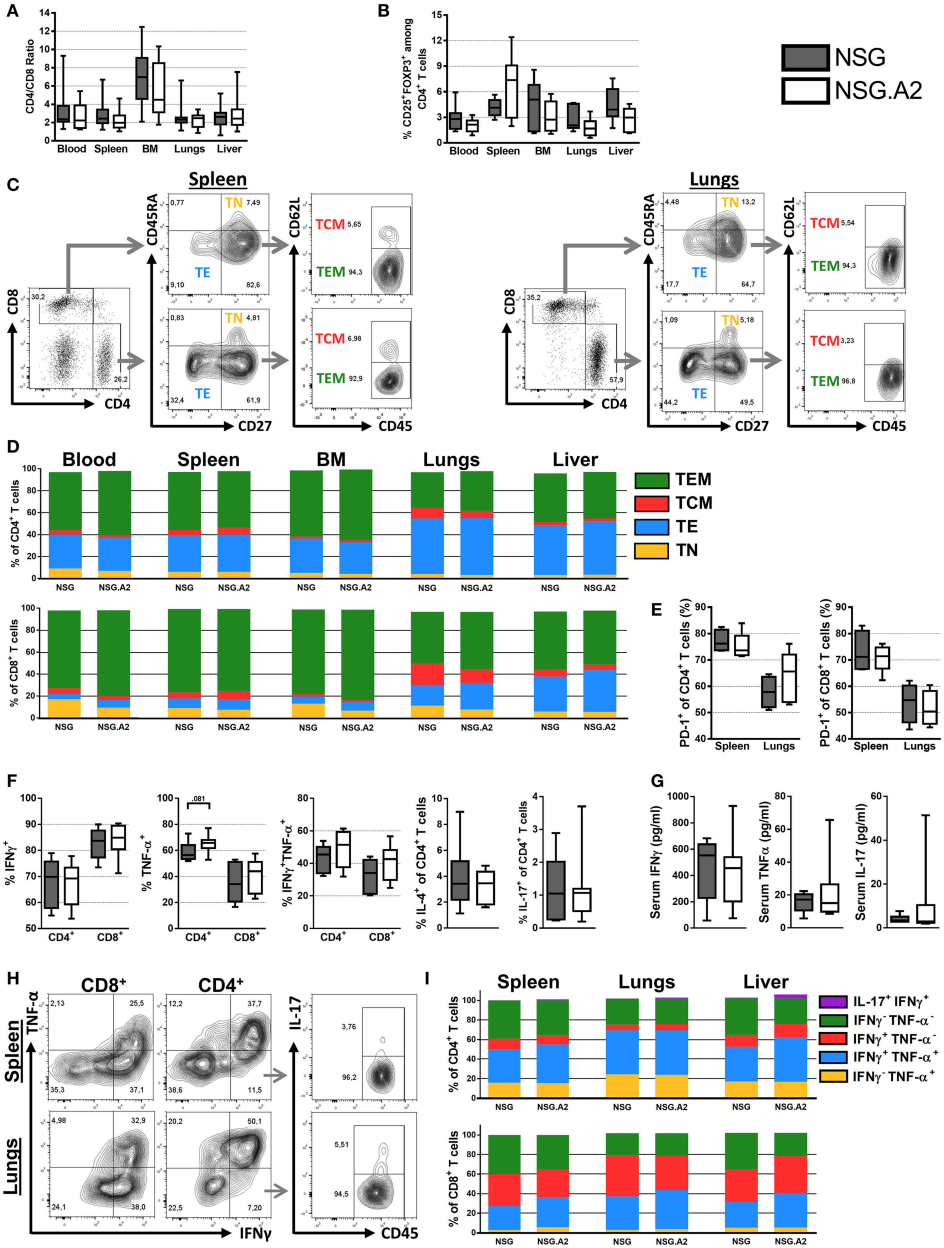
Differentiation and phenotype of human T cells in NSG and NSG-HLA-A2/HHD mice transplanted with non-HLA-A2 PBMCs. NSG and NSG-HLA-A2/HHD (NSG.A2 in figure) mice were transplanted with HLA-A2^−^ PBMCs and were sacrificed at day 14 to collect their organs and perform flow cytometry analyzes. **(A)** CD4/CD8 ratio in indicated organs (11 mice/group). **(B)** Frequency of Treg in the different organs (11 mice/group). **(C)** Representative FACS plots and gating strategy of the data shown in panel **D**. **(D)** Mean frequency of naive (TN, CD45RA^+^CD27^+^), effector (TE, CD45RA^−^CD27^−^), central memory (TCM, CD45RA^−^CD27^+^CD62L^+^) and effector memory (TEM, CD45RA^−^CD27^+^CD62L^−^) T cells (11 mice/group). **(E)** Frequency of PD-1^+^ T cells (4–5 mice/group). **(F)** Frequency of IFNγ^+^, TNF-α^+^, IFNγ^+^TNF-α^+^, IL-4^+^, and IL-17^+^ T cells in spleen (11 mice/group). **(G)** Serum concentration of IFNγ, TNF-α and IL-17 cytokines, assessed by bioplex (6–7 mice/group). **(H)** Representative FACS plots and gating strategy of the data shown in panel **I**. **(I)** Mean frequency of IFNγ^−^TNF-α^+^, IFNγ^+^TNF-α^+^, IFNγ^+^TNF-α^−^, IFNγ^−^TNF-α^−^, and IL-17^+^IFNγ^+^ T cells (4–5 mice/group). Data in **A, B, E, F**, and **G** show the median, 25 and 75th percentiles of the distribution (boxes), and whiskers extend to the 5 and 95th percentiles.

Median CD4/CD8 ratios were comparable in NSG and in NSG-HLA-A2/HHD mice and ranged between 2 and 3 in the blood, spleen, lungs and liver and were above 4 in the bone marrow (Figure 2A).

##### CD4^+^ T cells

The frequency of Treg among CD4^+^ T cells in the blood, spleen, bone marrow as well as in xGVHD target organs in both NSG and NSG-HLA-A2/HHD mice ranged between 12.4 and 0.6% (Figure 2B). The main subpopulations of CD4^+^ T cells in the blood, spleen and bone marrows were TEM, followed by TE while there was a relatively low frequency of TN and TCM (Figures 2C,D). Remarkably, the proportions of TE were significantly lower in the peripheral blood of NSG mice and NSG-HLA-A2/HHD mice than in their lungs (*P* = 0.002 and *P* < 0.001, respectively) or liver (*P* = 0.11 and *P* < 0.001, respectively), demonstrating a higher frequency of effector T cells in xGVHD-target organs (Figure 2D). Further, the proportions of CD4^+^ T cells expressing PD-1 were significantly higher in the spleen of NSG and NSG-HLA-A2/HHD mice than in their lungs (*P* = 0.03 and *P* = 0.09, respectively; Figure 2E).

Interestingly, the distribution of the different CD4^+^ T cell subtypes in the blood, spleen, lungs and liver were similar between NSG and NSG-HLA-A2/HHD mice.

In concordance with RNAseq analyses, assessment of the production of cytokine by splenic human T cells after stimulation by PMA/ionomycin demonstrated a TH1 profile (the majority of the cells were INFγ^+^ while very few CD4^+^ T-cells were IL-4^+^ or IL-17^+^; Figures 2F–I). Similarly, the majority of CD4^+^ T cells in xGVHD target organs from NSG and NSG-HLA-A2/HHD mice were positive for TNFα, INFγ or both (Figures 2H,I).

##### CD8^+^ T cells

The majority of CD8^+^ T cells present in NSG and NSG-HLA-A2/HHD mice were TEM (Figures 2C,D). As observed for CD4^+^ T cells, the proportions of CD8^+^ TE were significantly lower in the peripheral blood (5% in NSG mice and 6% in NSG-HLA-A2/HHD mice) than in the lungs [18% in NSG mice (*P* < 0.001) and 21% in NSG-HLA-A2/HHD mice (*P* = 0.003)] or the liver [22% in NSG mice (*P* < 0.001) and 35% in NSG-HLA-A2/HHD mice (*P* < 0.001)]. In contrast, there were significantly less naïve CD8^+^ T cells in the liver than in the blood of NSG (2 vs. 16%, *P* = 0.001) and NSG-HLA-A2/HHD mice (2 vs. 8%, *P* = 0.056). Further, the proportions of CD8^+^ T cells expressing PD-1 were significantly higher in the spleen of NSG (71%) and NSG-HLA-A2/HHD (71%) mice than in their lungs [55% for NSG mice (*P* = 0.03) and 50.4% for NSG-HLA-A2/HHD mice (*P* = 0.008); Figure 2E).

Interestingly, there was a higher proportion of naïve CD8^+^ T cells in the blood of NSG than in the blood of NSG-HLA-A2/HHD (16 vs. 8%, *P* = 0.007), suggesting higher CD8^+^ T-cell differentiation in NSG-HLA-A2/HHD mice (Figure 2D). In addition, there was a lower proportion of CD8^+^ T cells INFγ^neg^TNFα^neg^ in the liver of NSG-HLA-A2/HHD mice than in those from NSG mice (*P* = 0.016; Figure 2I).

##### Serum cytokine levels

In concordance with our flow-cytometry analyses, serum bioplex analyses revealed high concentration of INFγ and to a lesser extend of TNFα in the sera of each NSG and NSG-HLA-A2/HHD mice, while there was a low concentration of IL-17 (Figure 2G).

#### NSG and NSG-HLA-A2/HHD mice infused with HLA-A2 hPBMC

**Figure 3:**
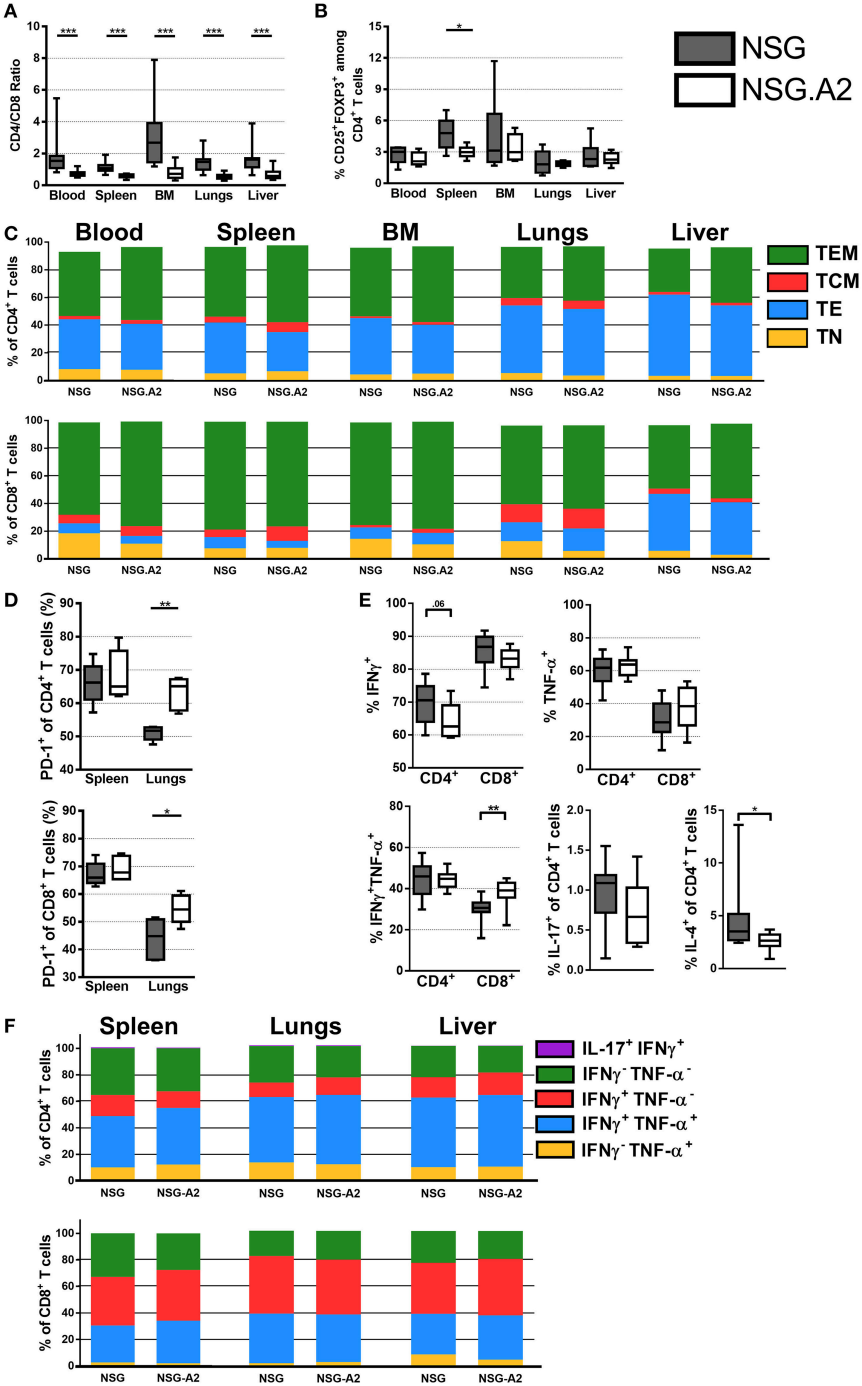
Differentiation and phenotype of human T cells in NSG and NSG-HLA-A2/HHD mice transplanted with HLA-A2 PBMCs. NSG and NSG-HLA-A2/HHD (NSG.A2 in figure) mice were transplanted with HLA-A2^+^ PBMCs and were sacrificed at day 14 to collect their organs and perform flow cytometry analyzes. **(A)** CD4/CD8 ratio in indicated organs (11–12 mice/group). **(B)** Frequency of Treg in the different organs (11–12 mice/group). **(C)** Mean frequency of naive (TN, CD45RA^+^CD27^+^), effector (TE, CD45RA^−^CD27^−^), central memory (TCM, CD45RA^−^CD27^+^CD62L^+^) and effector memory (TEM, CD45RA^−^CD27^+^CD62L^−^) T cells (11–12 mice/group). **(D)** Frequency of PD-1^+^ T cells in spleen (5 mice/group). **(E)** Frequency of IFNγ^+^, TNF-α^+^, IFNγ^+^TNF-α^+^, IL-4^+^, and IL-17^+^ T cells in spleen (11 mice/group). **(F)** Mean frequency of IFNγ^−^TNF-α^+^, IFNγ^+^TNF-α^+^, IFNγ^+^TNF-α^−^, IFNγ^−^TNF-α^−^, and IL-17^+^IFNγ^+^ T cells (5 mice/group). Data in **A, B, D**, and **E** show the median, 25 and 75th percentiles of the distribution (boxes), and whiskers extend to the 5 and 95th percentiles (*p < 0.05, **p < 0.005, ***p < 0.0005).

In contrast to what was observed with infusion of non-HLA-A2 PBMC, median CD4/CD8 ratios were significantly higher in NSG than in NSG-HLA-A2/HHD mice in each assessed organs when HLA-A2 hPBMC were infused (Figure 3A). With this exception, main findings seen with the infusion of non-HLA-A2 PBMC were also observed with the infusion of HLA-A2 PBMC: Treg frequencies within the normal range (Figure 3B), higher proportions of CD4^+^ and CD8^+^ TE in xGVHD target organs than in the blood (Figure 3C), lower PD-1 expression in the spleen than in the lungs (Figure 3D), and predominant Th-1 profile (Figures 3E,F).

Interestingly, there was a higher proportion of naïve CD8^+^ T cells in the blood (*P* = 0.027) and in the lungs (*P* = 0.01) of NSG than in the blood of NSG-HLA-A2/HHD, suggesting higher CD8^+^ T-cell differentiation in NSG-HLA-A2/HHD mice (Figure 3C). There was also a higher proportion of CD8^+^ TCM in the bone marrow of NSG-HLA-A2/HHD mice (*P* = 0.008).

### TCR repertoire of human T cells infiltrating the spleen and the lungs of NSG and NSG-HLA-A2/HHD mice

**Figure 4:**
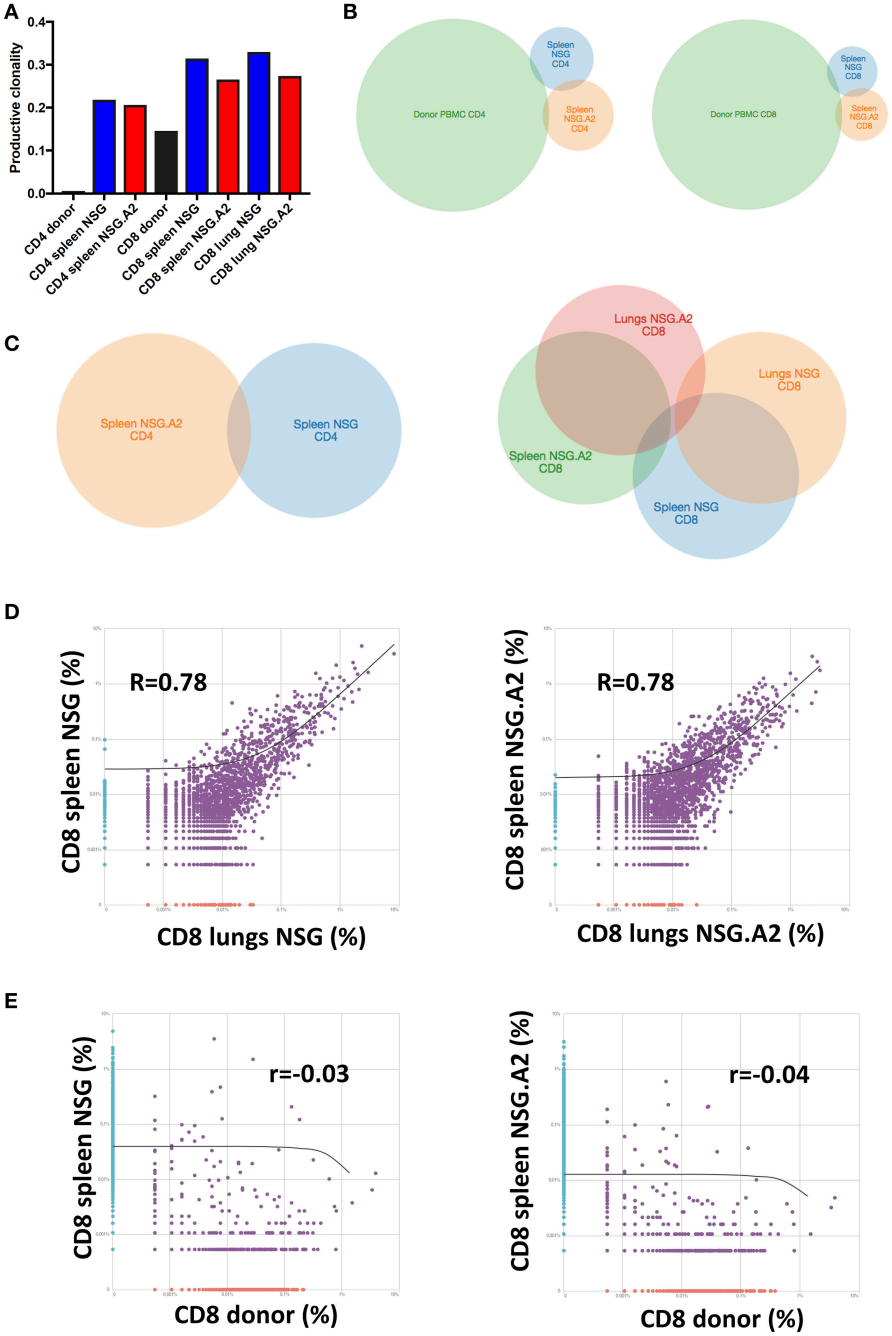
T-cell repertoire of CD4^+^ and CD8^+^ T cells in NSG and NSG-HLA-A2/HHD mice. NSG and NSG-HLA-A2/HHD (NSG.A2 in figure) mice were transplanted with HLA-A2^−^ PBMCs and were sacrificed at day 14 to perform TCRβ sequencing on T cells sorted and pooled (10 mice pooled/condition) from their spleen and lungs. **(A)** Productive clonality of CD4^+^ and CD8^+^ T cells sorted from donor PBMCs (before infusion to mice), spleen and lungs of NSG and NSG-HLA-A2/HHD mice. **(B,C)** Venn diagram comparing the identity of clonotypes between indicated samples **(D,E)** Correlations of clonotypes productive frequency between indicated samples.

Looking at the repartition of TCRβ families, as previously reported by us and by others in NSG mice using classical spectratyping technique (21, 22), TCRβ repertoire appeared highly polyclonal in the spleen and lungs of each NSG and NSG-HLA-A2/HHD mice (Supplemental Figure 1). However, specific TCR sequencing analyses demonstrated that the productive clonality (a normalized score based on diversity and sample entropy for which values near 0 represent more polyclonal samples and values near 1 represent more oligoclonal samples) was markedly higher (meaning less diversity) in each CD4^+^ and CD8^+^ T cells in the spleen on day 14 after transplantation than at the time of infusion. This evidences dramatic TCRβ repertoire narrowing after transplantation (Figure 4A).

As previously well-established, TCR diversity was lower in CD8^+^ than in CD4^+^ T cells at infusion (44), and this remained the case on day 14 after transplantation. Interestingly, the productive clonality was comparable in the spleen and in the lungs on day 14 after transplantation but was slightly lower (meaning more diversity) in NSG-HLA-A2/HHD than in NSG mice suggesting that additional antigens were recognized by donor CD8^+^ T cells in NSG-HLA-A2/HHD mice. In contrast, productive clonality was comparable in CD4^+^ T cells from NSG and NSG-HLA-A2/HHD mice (Figure 4A).

We next compared the identity of clonotypes between hPBMCs before infusion and in spleen post-infusion. As showed in the Figure 4B, we observed a very low overlap of their identity for each CD4^+^ and CD8^+^ T cells. This, together with the dramatic decrease of TCR diversity post-infusion, demonstrates that xGVHD is caused by a fraction of T cells having a low frequency in donor PBMCs (and therefore undetected in these samples) which subsequently expand in mouse organs.

We next compared the identity of clonotypes between organs and mouse types. As shown in the Figures 4C,D, we observed a low overlap of clonotype identity between the CD4^+^ and CD8^+^ T cells harvested from the spleen of NSG vs. NSG-HLA-A2/HHD mice. In contrast, there was some overlap between clonotypes identity observed in the spleens vs. the lungs in both mouse strains. Finally, no correlation of CD8^+^ T cell clonotypes frequency was observed between donor PBMCs and in spleen post infusion, either in NSG or NSG-HLA-A2/HHD mice (Figure 4E).

These data show that expression of HLA-A0201 impacted the repertoire of T cells causing xGVHD, and that a proportion of T cells with similar clonotypes were present in the spleen and xGVHD target organs.

### Survival and xGVHD scores

**Figure 5:**
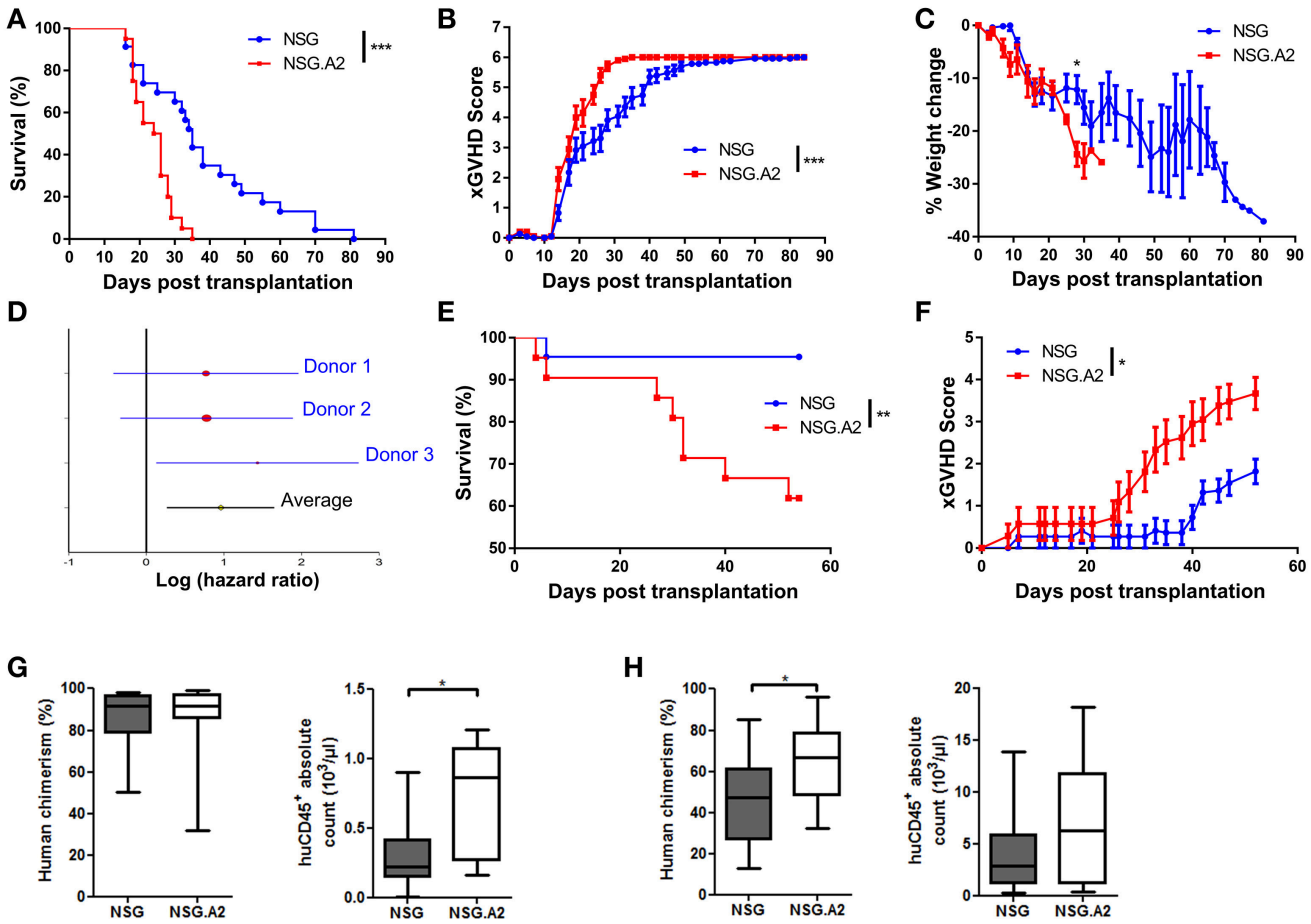
Higher lethality and severity of xGVHD in NSG-HLA-A2/HHD mice transplanted with non-HLA-A2 PBMCs. NSG and NSG-HLA-A2/HHD (NSG.A2 in figure) mice were transplanted with 2 × 10^6^ **(A–D)** or 1 × 10^6^ **(E,F)** HLA-A2^−^ PBMCs intravenously. **(A)** Pooled survival rates from the three different experiments [*n* = 23 (NSG) vs. 20 (NSG-HLA-A2/HHD)]. **(B,C)** Pooled xGVHD scores **(B)** and weight loss **(C)** from the three different experiments. **(D)** Meta-analyses of Hazard ratio obtained with each different donor. **(E,F)** Pooled survival rates and xGVHD scores from two different experiments (*n* = 22 (NSG) vs. 21 (NSG-HLA-A2/HHD). **(G)** Day 14 human cell chimerism and absolute counts in NSG (*n* = 11) and NSG-HLA-A2/HHD (*n* = 11) mice transplanted with 2 × 10^6^ PBMCs. **(H)** Day 14 human cell chimerism and absolute counts in NSG (*n* = 20) and NSG-HLA-A2/HHD (*n* = 13) mice transplanted with 1 × 10^6^ PBMCs. Data in **G** and **H** show the median, 25 and 75th percentiles of the distribution (boxes), and whiskers extend to the 5 and 95th percentiles (*p < 0.05, **p < 0.005, ***p < 0.0005).

We next investigated whether the expression of HLA-A0201 by NSG mice not only impacted the repertoire of T cells causing xGVHD but also exacerbated xGVHD. We first compared xGVHD in NSG and NSG-HLA-A2/HHD mice transplanted with 2 × 10^6^ hPBMCs from non-HLA-A2 donor after 2.5 Gy irradiation. As shown in Figures 5A–C, xGVHD lethality and severity were higher in NSG-HLA-A2/HHD than in NSG mice. The higher lethality was consistent in the three independent experiments and confirmed in a multivariate Cox regression model adjusted for weight, female PBMC donor to male mouse, and age of mice at transplantation (HR = 1.8, 95% CI 1.2–2.8, *P* = 0.007; Figure 5D). When mice were transplanted with 1 × 10^6^ hPBMCs, xGVHD lethality and severity were also significantly higher in NSG-HLA-A2/HHD than in NSG mice (Figures 5E,F).

**Figure 6:**
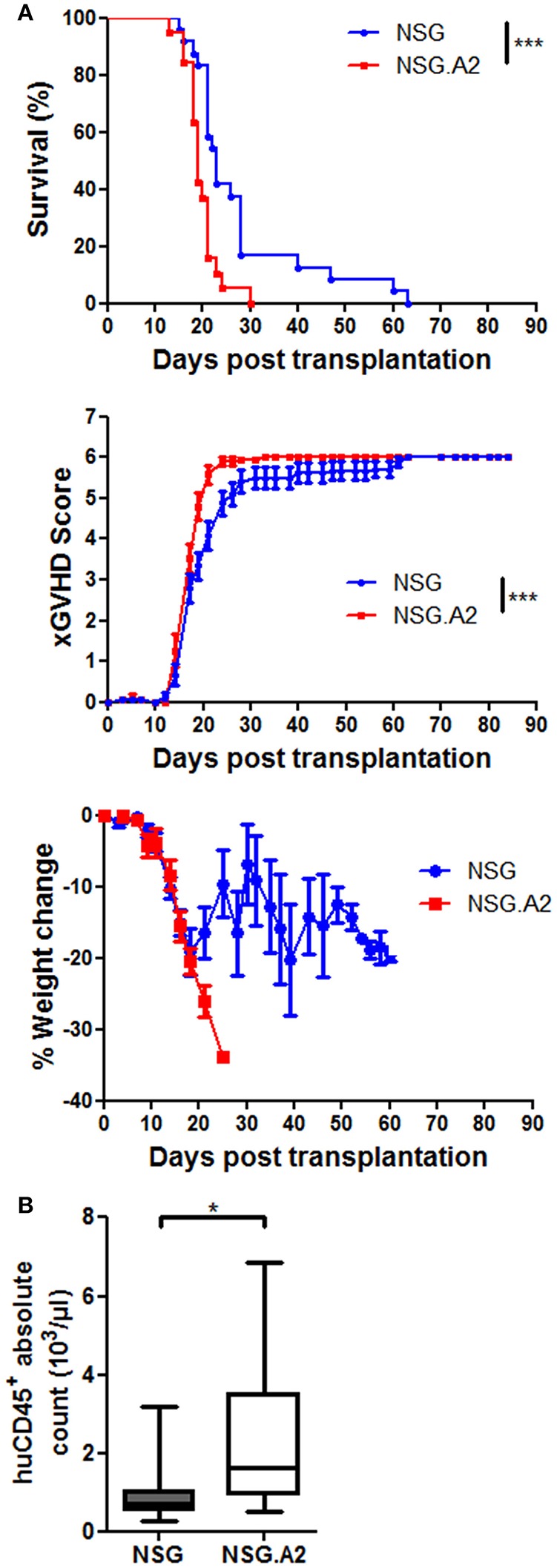
XGVHD in NSG-HLA-A2/HHD mice transplanted with HLA-A2^+^ donors. NSG and NSG-HLA-A2/HHD (NSG.A2 in figure) mice received sublethal total body irradiation (2.5 Gy) and were infused 24 h later with 2 × 10^6^ human PBMCs intravenously. PBMCs were isolated from HLA-A2^+^ healthy donors. **(A)** Survival, xGVHD scores and weight loss comparison of NSG and NSG-HLA-A2/HHD mice transplanted with three different HLA-A2^+^ donors (in three independent experiments). **(B)** Day 14 total cell count of human CD45^+^ cells in blood, calculated based on the absolute number of white blood cells (counted by using a Sysmex XS-800i cell counter) and on the frequency of human CD45^+^ cells among the total white blood cell population (%_human_CD45^+^ + %_mouse_CD45^+^). Data show the median, 25 and 75th percentiles of the distribution (boxes), and whiskers extend to the 5 and 95th percentiles (*p < 0.05, ***p < 0.0005).

We next hypothesized that in the case of NSG-HLA-A2/HHD mice injected with HLA-A2 hPBMC, human T cells could react against host cells expressing mouse peptide antigens bound to HLA-A2, as suggested by Patton et al. (45). We indeed observed a slightly more severe xGVHD in NSG-HLA-A2/HHD mice than in NSG mice after infusion of 2 × 10^6^ hPBMCs from HLA-A2 donors (Figure 6A).

#### Human cell expansion, infiltration and histologic xGVHD

In mice transplanted with 2 × 10^6^ hPBMCs from non-HLA-A2 donor, there were higher absolute counts of human hematopoietic cells in the blood of NSG-HLA-A2/HHD mice than in the blood of NSG mice (*P* = 0.041) while human chimerism levels were high (median > 90%) in each NSG and NSG-HLA-A2/HHD mice (Figure 5G). Comparable findings were made with HLA-A2 donors (Figure 6B). In mice transplanted with 1 × 10^6^ hPBMCs from non-HLA-A2 donor, there was a trend for higher absolute counts of human hematopoietic cells in the blood of NSG-HLA-A2/HHD mice than in the blood of NSG mice (*P* = 0.25), while human chimerism levels were significantly higher NSG-HLA-A2/HHD than in NSG mice *P* = 0.013; Figure 5H).

**Figure 7:**
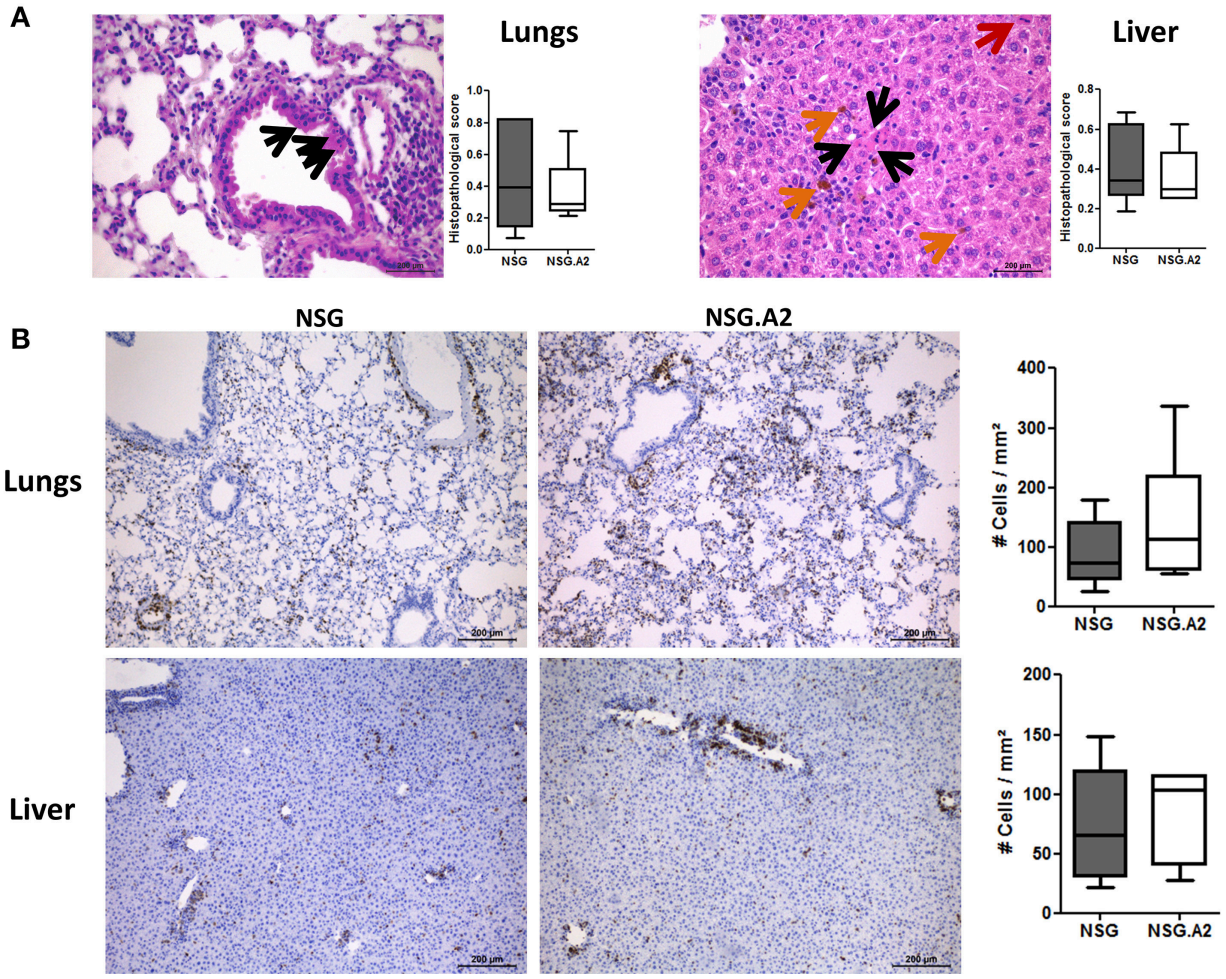
Histological analyses in NSG and NSG-HLA-A2/HHD mice transplanted with non-HLA-A2 PBMCs. NSG and NSG-HLA-A2/HHD (NSG.A2 in figure) mice received sublethal total body irradiation (2.5 Gy) and were infused 24 h later either with 2 × 10^6^ human PBMCs intravenously. PBMCs were isolated from HLA-A2^−^ healthy donors and experiment was repeated twice with different donors of PBMCs. Mice were sacrificed at day 14 to collect their organs. **(A)** Histopathological evaluation of GVHD score in lungs and liver. Black arrows show apoptotic bodies, red arrows show mitosis and orange arrows show bile plugs. Panels on the right show the comparison of 7–8 mice/group. **(B)** Histological quantification of human CD3^+^ cells (stained in brown) infiltration in lungs and liver of NSG and NSG-HLA-A2/HHD mice. Panels on the right show the comparison of 7–8 mice/group. Data show the median, 25 and 75th percentiles of the distribution (boxes), and whiskers extend to the 5 and 95th percentiles.

**Figure 8:**
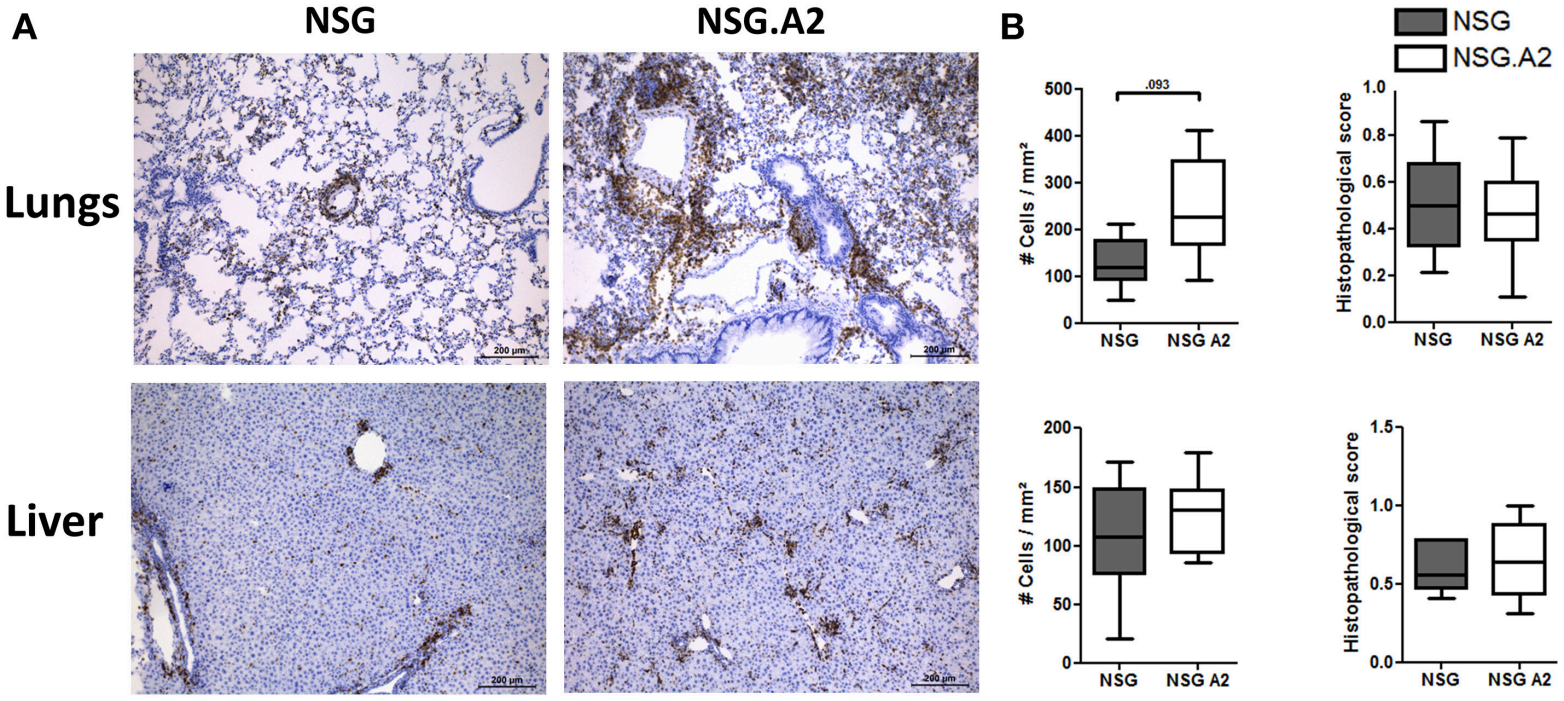
Histological analyses in NSG and NSG-HLA-A2/HHD mice transplanted with HLA-A2 PBMCs. NSG and NSG-HLA-A2/HHD (NSG.A2 in figure) mice received sublethal total body irradiation (2.5 Gy) and were infused 24 h later with 2 × 10^6^ human PBMCs intravenously. PBMCs were isolated from HLA-A2^+^ healthy donors. Mice were sacrificed at day 14 to collect their organs. **(A)** Histological quantification of human CD3^+^ cells (stained in brown) infiltration in lungs and liver of NSG and NSG-HLA-A2/HHD mice. Panels on the right show the comparison of 7–8 mice/group. **(B)** Histopathological evaluation of GVHD score in lungs (top panel) and liver (bottom panel). Data show the median, 25 and 75th percentiles of the distribution (boxes), and whiskers extend to the 5 and 95th percentiles.

Typical histologic signs of GVHD (such as apoptotic bodies or bile plugs) were found in the liver and lungs of each NSG and NSG-HLA-A2/HHD mice while histological GVHD scores were comparable in NSG and NSG-HLA-A2/HHD mice irrespective of the HLA-A2 positivity or not of the donor (Figures 7, 8).

### GvL effects in NSG and NSG-HLA-A2/HHD mice

**Figure 9:**
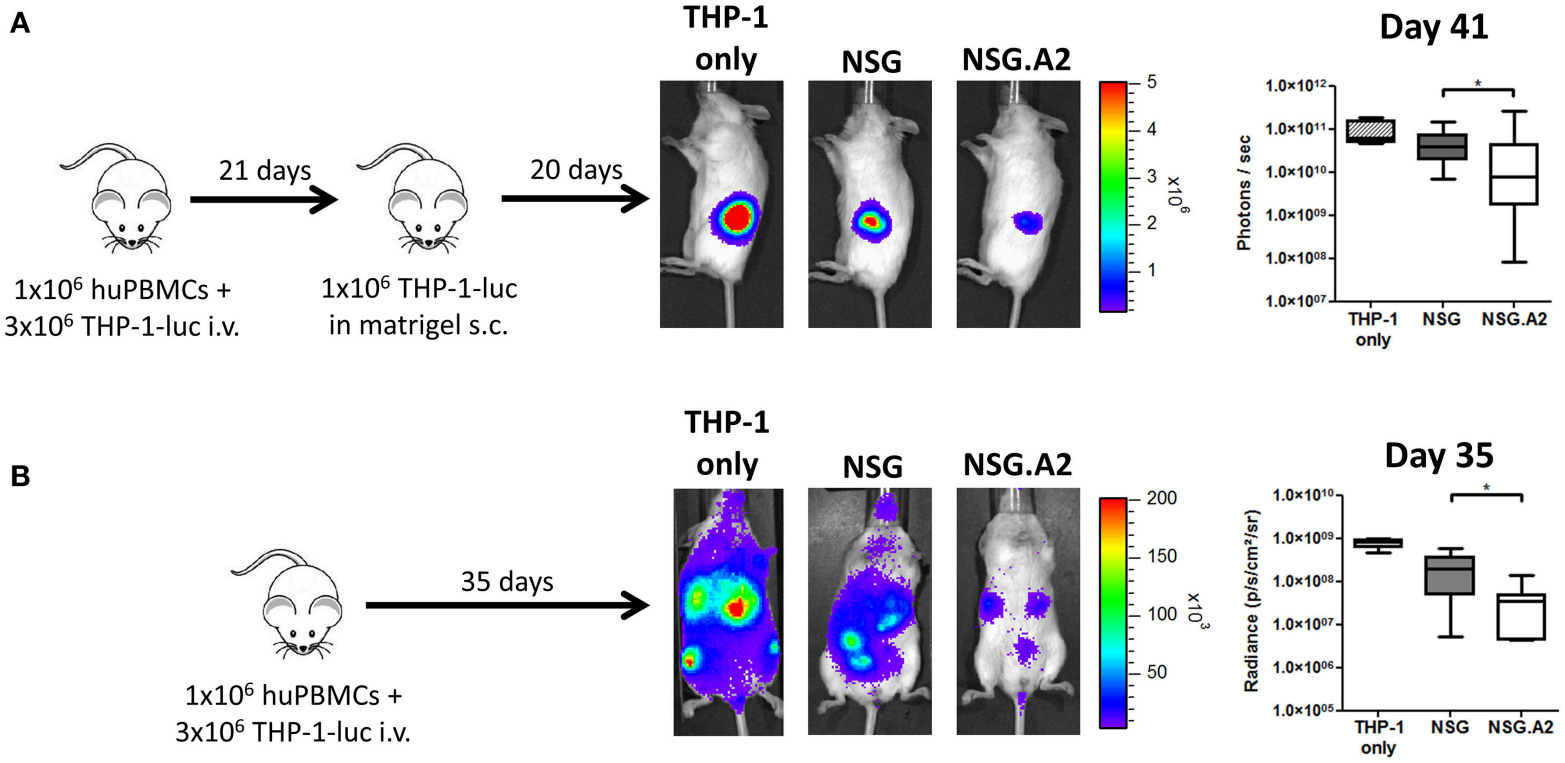
Higher graft-versus-leukemia effect in NSG-HLA-A2/HHD mice. NSG and NSG-HLA-A2/HHD (NSG.A2 in figure) mice received sublethal total body irradiation (2.5 Gy) and were infused 24 h later with 1 × 10^6^ of human PBMCs and 3 million of THP-1-luc cells intravenously. **(A)** At day 21, all the mice transplanted at day 0 (14 NSG and 13 NSG-HLA-A2/HHD) as well as four additional untransplanted NSG mice received a subcutaneous injection of 1 million of THP-1-luc cells in matrigel. Experimental design, representative images of bioluminescence at day 41 post-transplantation and comparison of luminescence are shown. **(B)** In a repetition of the first experiment, mice received only the intravenous injection of THP-1-luc cells [*n* = 4 (THP-1 only), 9 (NSG) and 8 (NSG-HLA-A2/HHD)]. Experimental design, representative images of bioluminescence at day 35 post-transplantation and comparison of luminescence are shown. Actual images of one representative mouse from each group are shown with Y-axis indicating photon flux (photons/s). Region of interest were drawn over the entire tumors **(A)** or over the entire body of each mouse **(B)**. Data show median values with interquartile range (*p < 0.05).

We finally assessed whether the more severe xGVHD observed in NSG-HLA-A2/HHD mice translated to higher GvL effects. Since THP-1 cells are HLA-A0201 positive (46, 47), we performed these experiments with hPBMC from non-HLA-A2 donor in order to have a similar target for xGVHD and GvL. In brief, NSG and NSG-HLA-A2/HHD mice were co-transplanted with 3 million of THP-1 cells, transfected to express the luciferase gene, and 1 million of human PBMCs (Figure 9A). Because very low levels of bioluminescence were observed at day 20, probably due to the elimination of THP-1 cells by hPBMCs, mice received a second injection of THP-1-luc cells, this time administered subcutaneously in matrigel, at day 21. As control, three additional NSG mice also received this subcutaneous injection without having received PBMCs or THP-1 cells previously. Twenty days later, bioluminescence was again monitored and we could observe that the tumor burden was significantly reduced in NSG mice transplanted with PBMCs in comparison with control mice, showing that human PBMCs eliminated THP-1 cells subsequently to GvL effects. Interestingly, the tumor burden was even more reduced in NSG-HLA-A2/HHD mice.

In a second experiment, and because luminescence of THP-1 cells could be still observed at day 20, we did not transplant additional THP-1 cells subcutaneously at day 21 (Figure 9B). At day 35, we observed an overall reduction of bioluminescence in NSG-HLA-A2/HHD mice in comparison with NSG mice, confirming the result of the first experiment.

## Discussion

The aim of the current study was to further describe the pathogenesis of xGVHD induced by injection of hPBMCs in classical NSG mice and to assess the impact of the expression of HLA-A0201 by NSG mice cells on xGVHD and GvL effects. For the later, we reasoned that the expression of HLA-A0201 by mouse cells might serve as an allogeneic antigen (in addition to xenogeneic antigens) in case of transplantation with cells from non-HLA-A0201 donor and might better present mouse peptide antigens bound to HLA-A2 to T cells from HLA-A0201 hPBMC donors. In addition, in case of assessment of GvL effects against the leukemic THP-1 cells, there were major antigens (HLA-A0201) shared by mouse and human leukemic cells in case of transplantation of hPBMC from a non-HLA-A2 donor, mimicking what is happening in patients in whom GVHD and GvL effects are frequently mediated in part toward similar antigens (48).

Using RNAseq analyses on T cells harvested on day 7 after transplantation we observed some similar gene activation patterns in T cells injected in mice and in T cells stimulated *in vitro* with CD3/CD28. This observation explains prior reports demonstrating that lack of expression of murine MHC-class 1 or MHC-class 2 dramatically decreased T-cell proliferation (20), while co-stimulation blockade with CTLA-4-Ig administration inhibited T-cell expansion and prevented/treated xGVHD, demonstrating that CD28–CD80/CD86 interactions were necessary for xGVHD (22). In addition to upregulated expression of genes involved in cell proliferation, as well as in TCR, co-stimulatory, IL-2/STAT5, PI3K/AKT/mTOR signaling (49), and Aurora kinase A pathway RNAseq analyses demonstrated the upregulation of TH1 (but not TH17) genes in T cells harvested in NSG mice in comparison to baseline T cells. Importantly, these data correlate well with a recent transcriptomic study performed in non-human primate demonstrating that alloreactive T cells in acute GVHD are specifically characterized by (i) the overexpression of Aurora kinase A, (ii) the upregulation of pathways implicated in proliferation and effector function, and (iii) a Th1-over-Th17-skewed immune response (43). The relevance of aurora kinase A in xGVHD of NSG mice is further supported by another recent study showing that its inhibition leads to a significant amelioration of survival (50). Altogether these data support the huPBMCs-infused NSG mice as robust model of human GVHD physiopathology.

Flow cytometry analyses of T cells harvested from the spleen of NSG and NSG-HLA-A2/HHD mice were consistent with RNAseq data and showed that, on day 14 after transplantation, most T cells were either TEM or TE (in concordance with the upregulation of the genes implicated in activation/proliferation observed in RNAseq analyses) and produced the TH1 (or TC1) cytokines INFγ and TNFα (or both). This observation is in line with prior reports showing the efficacy of therapy blocking TNFα for xGVHD prevention in NSG mice (20), despite a recent study unraveled a “Treg promoting role” for TNFα trough TNFR2 stimulation (51). Also, in concordance with the lack of Th17 gene upregulation observed in RNAseq analysis in T cells harvested from mice spleen on day 7, day 14 flow cytometry analyses demonstrated that only a low percentage of CD4^+^ T cells expressed IL-17. Further, most of these CD4^+^IL-17^+^ T-cells also secreted INFγ.

In concordance with the observation that T-cell proliferation in both NSG and NSG-HLA-A2/HHD mice is dependent on TCR signaling, specific clonotypes analyses demonstrated dramatically decreased TCR diversity in CD4^+^ and CD8^+^ T cells harvested in the spleen or lungs of NSG or NSG-HLA-A2/HHD mice on day 14 after transplantation in comparison to TCR diversity at infusion. Further, there was a very low overlap for each CD4^+^ and CD8^+^ T cells between clonotypes at infusion and after transplantation. This demonstrates that xGVHD is caused by T cells having a low frequency in donor PBMCs (and therefore mostly undetected in these samples) which subsequently expand in mice.

Interestingly, there was a good correlation between the frequencies of specific CD8^+^ T-cell clones in the spleen and in the lungs on day 14 after infusion. This suggests that the xGVHD reaction is at least in part systemic and not (always) restricted to one specific organ. Interestingly, the CD8^+^ T cell TCR diversity was higher in NSG-HLA-A2/HHD than in NSG mice suggesting that additional antigens were recognized by donor CD8^+^ T cells in NSG-HLA-A2/HHD (in comparison to NSG) mice. Despite the significant correlation between specific CD8^+^ T-cell clonotype frequencies in the spleen and in the lungs of treated animals, we observed that the T-cell sub-phenotypes were distinct in the spleen and in xGVHD target organs. Specifically, T cells infiltrating the lungs or the liver had more frequently a TE but less frequently a naïve phenotype than those from the spleen.

In addition to its impact on T-cell repertoire, we observed that HLA-A0201 expression by NSG promoted CD8^+^ T-cell expansion and exacerbated xGVHD. These results are in line with a prior observation showing that after injection of purified hematopoietic stem cells, NSG-HLA-A2/HHD but not NSG mice developed xGVHD (45). Finally, as observed in humans in which there is a strong correlation between GVHD and GvL effects, we observed enhanced GvL effects in NSG-HLA-A2/HHD mice. Altogether, these results show that HLA-A0201 molecules participate to the presentation of antigens to allogeneic T cells and therefore that hPBMC-infused NSG-HLA-A2/HHD mice could serve as model of combined xeno and allogeneic GVHD.

In conclusion, our data demonstrate that the pathogenesis of xGVHD shares important features with human GVHD such as TCR/co-stimulatory-mediated expansion of selected T-cell clones that acquire mainly a Th1/Tc1 profile (52). This strengthens the robustness of this platform to study the impact of various therapeutic agents on GVHD. Further, our data show that NSG-HLA-A2/HHD mice could serve as model of combined xeno and allogeneic GVHD/GvL effects.

## Author contributions

GE, MH, LD, GF, CR, LB, and JM performed the experiments; GE, JS, H-JW, SH-B, and FB analyzed and interpreted the data; PD, YB and HL helped in data interpretation; GE, SH-B, and FB designed the research; GE and FB wrote the article and all authors edited the manuscript.

### Conflict of interest statement

The authors declare that the research was conducted in the absence of any commercial or financial relationships that could be construed as a potential conflict of interest.
