# Supplementary material for: Xenogeneic Graft-Versus-Host Disease in Humanized NSG and NSG-HLA-A2/HHD Mice
Source: Front Immunol. 2018 Aug 30;9:1943. doi: 10.3389/fimmu.2018.01943 (PMC6125392; doi:10.3389/fimmu.2018.01943)
Supplement: Supplementary file 8 [file Image_1.PDF]

A

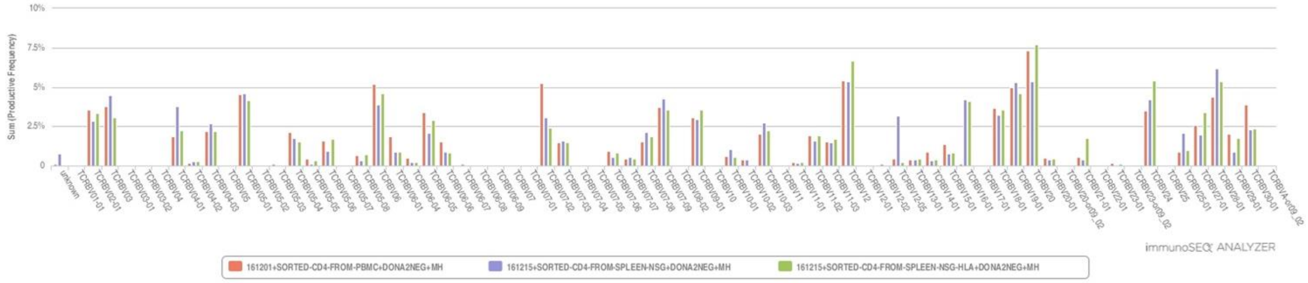

B

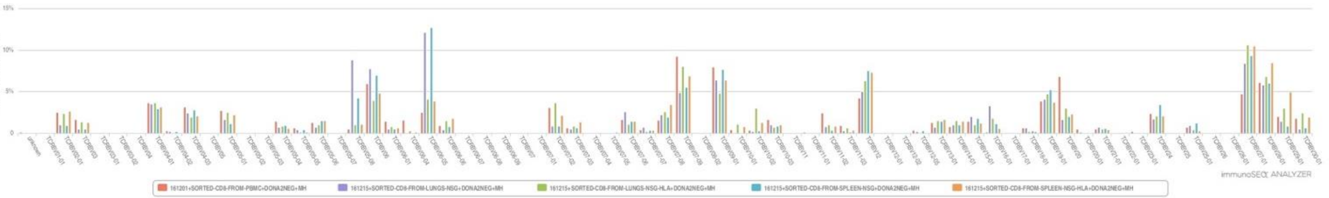

Supplemental Figure 1. Ten NSG and NSG-HLA-A2/HHD mice received sublethal total body irradiation (2.5 Gy) and were infused 24h later with 2x10^{6} human PBMCs intravenously. PBMCs were isolated from an HLA-A2^{-} healthy donor. Mice were sacrificed at day 14 to collect their spleen and lungs. Spleen and lungs homogenates were respectively pooled (10 NSG and 10 NSG.A2) and 1 million of CD4^{+} and CD8^{+} T cells were sorted by flow cytometry. DNA was extracted immediately after sorting and TCRB sequencing was performed by Adaptive Biotechnologies. (A-B) TCRB V family usage of CD4^{+} T cells in donor PBMCs (orange) and spleen of NSG (purple) and NSG-HLA-A2/HHD mice (green) (A) and of CD8^{+} T cells in donor PBMCs (orange), lungs of NSG (purple) and NSG-HLA-A2/HHD (green) and in spleen of NSG (blue) and NSG-HLA-A2/HHD mice (orange) (B).
